# Sexual Dimorphism: The Interrelation of Shape and Color

**DOI:** 10.1007/s10508-024-02918-1

**Published:** 2024-06-28

**Authors:** Šimon Pokorný, Ondřej Pavlovič, Karel Kleisner

**Affiliations:** Department of Philosophy and History of Science, Faculty of Science, Charles University, Albertov 6, 128 00 Prague, Czech Republic

**Keywords:** Sex typicality, Sexual shape dimorphism, Skin color, Facial contrast

## Abstract

Sex-typicality displayed as sexual dimorphism of the human face is a key feature enabling sex recognition. It is also believed to be a cue for perceiving biological quality and it plays an important role in the perception of attractiveness. Sexual dimorphism of human faces has two main components: sexual shape dimorphism of various facial features and sexual color dimorphism, generally manifested as dimorphism of skin luminance, where men tend to be darker than women. However, very little is known about the mutual relationship of these two facets. We explored the interconnection between the dimorphism of face shape and dimorphism of face color in three visually distinct populations (Cameroonian, Czech, and Vietnamese). Our results indicated that populations which showed a significant dimorphism in skin luminance (Cameroon, Vietnam) had low levels of sexual shape dimorphism, while a population with higher levels of sexual shape dimorphism (Czech Republic) did not exhibit a significant dimorphism of skin luminance. These findings suggest a possible compensatory mechanism between various domains of sexual dimorphism in populations differing in the levels of shape and color dimorphism.

**Supplementary Information:**

The online version contains supplementary material available at 10.1007/s10508-024-02918-1.

## Introduction

The face is usually the most frequently displayed part of the human body. It presents visual cues for age (Porcheron et al., 2017), attractiveness (Rhodes, 2006), health (Henderson et al., 2016), individual identity (Sheehan & Nachman, 2014), as well as sexual identity (Roberts & Bruce, 1988; Bruce & Langton, 1994; Komori et al., 2011). The perception of such cues is fast and accurate (Bruce & Young, 1986; O’Toole et al., 1998), enabling us to form first impressions about the unknown person. Although sexual identity can also be assessed from a range of other cues, ranging from visible primary sexual features and overall body shape to cultural features such as clothing, hairstyle, and jewelry, facial sexual dimorphism is by itself sufficient for sex classification (Cellerino et al., 2004).

Reported accuracy of sex recognition by human raters varies depending on study design but is overall very high, ranging from 96% (Burton et al., 1993) to 100% (Bruce & Young, 1986) in adult participants. Infants and children tend to be less accurate (Wild et al., 2000), which indicates that this ability is acquired and honed throughout development. Still, the core of this process is as yet not well understood. It has been reported that some particular features (eyes, nose) facilitate sex recognition (Roberts & Bruce, 1988), but by themselves, they do not suffice for classification (Brown & Perrett, 1993; Bruce et al., 1993). Dupuis-Roy et al. (2009) have identified some areas important for sex classification, such as the eye area and mouth area, but in the absence of clear cues, such as red lips, the participants compensated by relying on different areas. This shows that this process combines information from multiple sexually dimorphic features.

Male and female faces differ in their overall shape and composition of features. The sum of these differences is measured as sex-typicality, usually quantified as Sexual Shape Dimorphism (SShD). Sexual dimorphism has been thoroughly studied in terms of femininity and masculinity of the human face (Kleisner et al., 2021; Komori et al., 2011; Mitteroecker et al., 2015; O’Toole et al., 1998). SShD can be partly explained by allometry (Kleisner et al., 2021; Zaidi et al., 2019) because men are overall taller and heavier than women. Other features (such as the size and shape of the jaws, cheekbones, lips, eyes, and nose) may be influenced by gonadal hormone levels (Fink et al., 2005; Thornhill & Gangestad, 1999), and could serve as a signal of immunocompetence (Perrett et al., 1998). These morphological differences are believed to be further enhanced by sexual selection (Claes et al., 2012; Marcinkowska et al., 2019), where more sex-typical faces are perceived as more attractive. However, more recent studies have challenged this idea (Jones et al., 2021; Gonzalez-Santoyo et al., 2015), finding little evidence of a relationship between immunocompetence and perceived attractiveness. Finally, there appears to be no association between immunocompetence and SShD either, apart from what can be explained by allometry (Zaidi et al., 2019).

The relationship between SShD and perceived attractiveness is also not straightforward (Kleisner et al., 2021). Rated attractiveness of women’s faces seems to be generally positively associated with their femininity (Perrett et al., 1998; Rhodes, 2006), although it is more closely affected by direct signals of biological quality, such as age and skin texture (Jones et al., 2004), but female preference for masculine male faces is at best inconsistent. Some studies have reported no effect of masculine appearance (Rhodes et al., 2003) or even preference for feminine male faces (Perrett et al., 1998). Other research suggests that preference for facial masculinity varies depending on changes in hormone levels during the menstrual cycle (Johnston et al., 2001; Jones et al., 2005; Penton-Voak & Perrett, 2000), but that was recently challenged by studies which reported no effect of women’s monthly hormonal fluctuations on their preference for male faces (Jones et al., 2018; Marcinkowska et al., 2018). Furthermore, while masculinity (maleness) as measured by geometric morphometrics does express facial sex-typicality, facial masculinity as perceived by human observers is associated with sexual dimorphism only in part (Mitteroecker et al., 2015).

An additional rationale behind male-specific characteristics may stem from intrasexual competition among males for mating opportunities (Puts, 2010). Male faces are generally wider to accommodate for bigger jaw and adjacent musculature, thereby offering enhanced resistance against blunt force trauma, such as would occur during male fights (Carrier & Morgan, 2015). This is further supported by the high accuracy of aggressiveness assessment and its correlation with fighting performance among males, reported by Třebický et al. (2013).

The role of masculine and feminine appearance in attractiveness rating and mate choice thus remains a topic of discussion, with some studies suggesting no association at all (Nakamura & Watanabe, 2020). Sex-typicality could, however, have an adaptive value on its own through the process of sex recognition. In a study by Hoss et al. (2005), sex classification of male faces was facilitated by masculinity in both adult and child raters. Sex classification of female faces, on the other hand, was facilitated by attractiveness, not by femininity. Femininity and masculinity are not two sides of the same coin: femininity is vaguely linked to attractiveness perception and could serve as an indirect cue to biological quality, while masculinity could be an adaptive cue for sex recognition and fighting ability but is not directly associated with attractiveness.

Apart from shape, male and female faces also vary in skin color: women have in general lighter skin than men do (Jablonski & Chaplin, 2000; van den Berghe & Frost, 1986; Wee et al., 2013). This type of sexual dimorphism has been attributed either to sexual selection (van den Berghe & Frost, 1986; Vera Cruz, 2018), with a prevalence of preference for lighter females among men, or to different needs for vitamin D_3_ (Jablonski & Chaplin, 2013). This sex-related difference is deeply ingrained in the human perception of male or female facial appearance (Frost, 1990). Even this skin color dimorphism could, however, be a result of intra-sexual, rather than inter-sexual selection: a study on Mexican men showed that darker skin tones were associated with higher dominance ratings but lower attractiveness (Martínez-Ramírez et al., 2023), pointing to its possible role in male competition.

Other studies described sexual differences in the luminance contrast between certain facial features (eyes, eyebrows, lips) and the surrounding skin: women have a generally higher facial contrast in the eye and mouth regions and, consequently, faces with a higher contrast are perceived as more feminine (Russell, 2003, 2009). In our previous study, we argued that facial contrast is a cue to perceiving facial skin color, which is why this effect could be a by-product of the overall dimorphism in skin color (Pokorný & Kleisner, 2021). Some recent studies moreover explored not only skin lightness (or darkness) but also various color hues. In a study including multiple ethnic groups, Wee et al. (2013) reported a sex difference in skin yellowness: they found that men are yellower than women. Men are also believed to be on average redder than women (Nestor & Tarr, 2008), although this is not supported by the results of Wee et al.

In the current study, we decided to investigate skin luminance and color through direct measurement as well as computation of facial contrast, which enables identification of even small differences. Measurable differences in skin luminance, color, and facial contrast in both luminance and color then all contribute to the overall Sexual Color Dimorphism (SCoD).

Sexually dimorphic traits are not universal across human populations (Claes et al., 2014). Recently, Kleisner et al. (2021) described the range and pattern of SShD in a number of visually distinct populations and concluded that it varies dramatically in populations from Africa, Europe, and South America. In our previous study, we found differences in facial contrast dimorphism in populations from Africa and Europe (Pokorný & Kleisner, 2021). Furthermore, a study by Fiala et al. (2022) found no set of universal sexually dimorphic cues that would predict perceived sex-typicality in both Africans and Europeans. Certain populations in the African Sahel/Savannah belt undertaking different subsistence strategies diverged in their sexually specific traits, but the same magnitude of sexual dimorphism persists across all populations (Kleisner et al., 2023). These findings have direct consequences for the study of sex recognition, because the levels and types of sexual dimorphism differ among populations.

Differences in color and contrast can facilitate sex recognition (Russell, 2009) but on their own, they are not sufficient for effective classification. The same applies to facial dimorphism: Burton et al. (1993) have shown that sex classification using only shape is possible but far less accurate than when information about texture is also available. Although certain traits tend to facilitate sex recognition under normal conditions, human raters can effectively use other sex differences when information from these primary traits is unavailable. In a study conducted by Hill et al. (1995), participants relied more on color information in a frontal view and on shape information in a lateral view. Yip and Sinha (2002) observed that when shape information is degraded, color cues become useful in identity recognition. In sex recognition, raters rely on the mouth area as long as it is informative, but when that information is insufficient, they switch to the eye area (Dupuis-Roy et al., 2009). This polymodal focus on face perception is key to understanding how human populations can vary so much in sexual dimorphism yet perform well on cross-cultural sex recognition.

Our aim here is to expand these conclusions and explore the relationship between the shape component (SShD) and the color component (SCoD) in samples of Asian (Vietnam), European (Czech Republic), and African (Cameroon) populations. Sexual dimorphism of the human face consists of several different components: (1) allometry, as a byproduct of the overall sexual dimorphism in body mass, (2) sex-typicality, as a means of sex-recognition, (3) other sex-specific signaling relevant for sexual selection, such as attractiveness in mate choice or dominance in male competition. These components manifest in either the shape or the color dimorphism, potentially both, and are subject to diverse selective pressures and environmental constraints. We, therefore, expect to find a different pattern of sexual dimorphism in each of the studied populations.

## Method

### Participants

Participants were recruited via social networks, flyers, or personally. All individuals involved in this study provided informed consent. Cameroonian participants were mostly students from the University of Buea, Czech participants were from Charles University in Prague, and Vietnamese participants were students at the University of Science and Technology in Hanoi. Participants who did not follow through the entire process of data collection were removed from the study and were not taken into account in the presented analysis.

The final dataset consisted of facial photographs of 91 Vietnamese participants (60 men, 31 women, M age ± SD = 21.42 ± 3.08), 98 Czechs (50 men, 48 women, M age ± SD = 23.93 ± 4.11), and 113 Cameroonians (50 men, 63 women, M age ± SD = 21.74 ± 3.09).

### Measures and Procedure

#### Photograph Acquisition

Facial portraits were acquired using a standardized procedure (Třebický et al., 2016). Participants’ portraits were taken in front of a white background with color camera Canon 60D using a studio electronic flash. For Vietnamese participants, we used the Canon RF 50 mm STM lens and the focus point was set to the left eye. Exposure was set to ISO 100, shutter speed to 1/160s, aperture f/8, and strobe was set to 2/3 power. Photographs were taken from a tripod set to match the sitting height of each participant, so that the target’s face was in the middle of the frame. Distance between the lens and the target’s tip of the nose was set for each individual to 125 cm to preserve the natural variability in facial size in each image and to obtain the sharpest possible result with the 50 mm lens. Participants sat on a chair with no backrest and were instructed to sit straight, adopt a neutral facial expression, and look directly into the camera. They were also asked to refrain from any facial makeup, glasses, jewelry, or other decorations. To eliminate the effect of varying clothing, all participants were dressed in plain black T-shirts.

Photographs were shot into uncompressed raw files (*.CR2 format), and later processed into JPEG files in sRGB color space in Adobe Photoshop Lightroom 4. At the beginning of each session, a white balance patch was photographed, and color calibration, exposure, and white balance performed using X-Rite Color Checker targets. The photographs were post-processed in Photoshop CS6: images were cropped so that participants’ faces were in all images in the same absolute position.

#### Color and Contrast Measurements

**Fig. 1:**
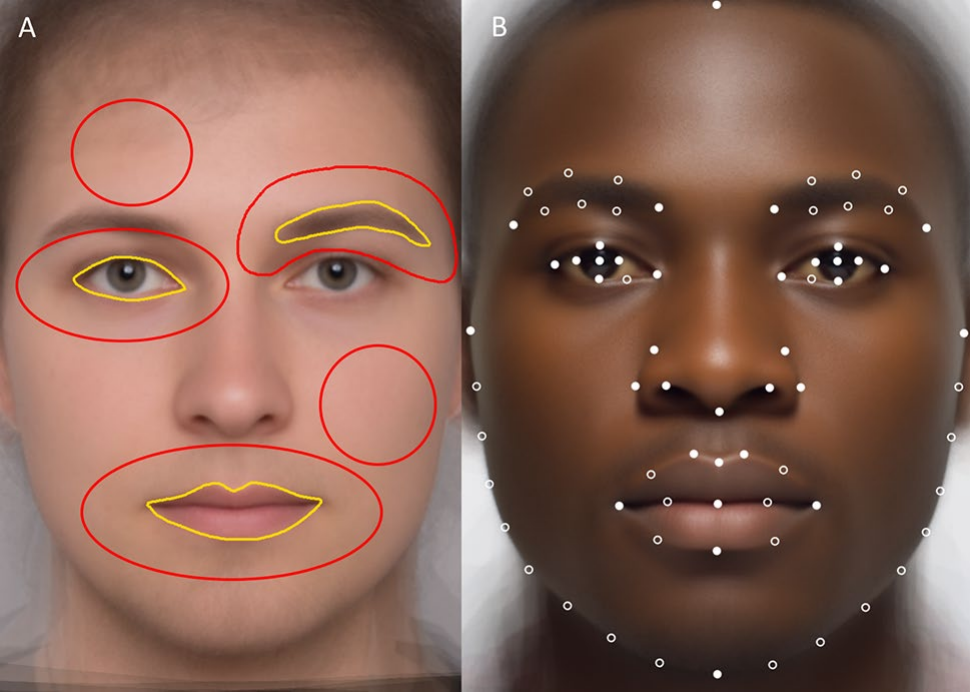
Facial color and shape measurements. **A** Feature and skin area selection: yellow lines show how features (lips, eyes, and brows) were selected, while the red lines show how the areas of surrounding skin, cheek, and forehead were selected. **B** Locations of 36 landmarks (full dots) and 36 semi-landmarks (circles). This image presents artificial faces (Color figure online)

In the next step, we applied the Color Transformer 2 plugin in ImageJ. Eye, lips, brows, and surrounding skin areas were selected using the freehand selection tool (Fig. 1), while skin patches from the forehead and the right cheek were selected using the oval selection tool. Mean luminance and color of the selected areas were measured in CIE L*a*b* color space. We used five measured skin areas (eye area, brow area, lips area, cheek, and forehead) to calculate mean lightness (L*), redness (a*), and yellowness (b*) of the skin.

Facial contrast in luminance, red, and yellow was calculated using adapted Michelson contrast (Russell, 2009) calculated as *C*_f_ = (*L*_s_ − *L*_f_)/(*L*_f_ + *L*_s_), where *L*_f_ stands for the mean feature (eye, brow, lips) color, *L*_s_ stands for mean skin color, and *C*_f_ is the feature/skin contrast. Resulting values can range between − 1 and 1: 0 indicates no contrast, values above 0 indicate that the feature is darker/greener/bluer than the skin, while values below 0 indicate that the feature is lighter/redder/yellower than the skin.

#### Geometric Morphometrics

Facial shape was digitized using tpsDig2 version 2.30 (Rohlf, 2015). We used a standard set of 72 landmarks (36 true landmarks, 36 semilandmarks) placed on a frontal facial image (Kleisner et al., 2010, 2019). Landmark configuration was symmetrized and subjected to Procrustes superimposition (GPA) using the *gpagen* function of the geomorph package in R (Adams & Otárola-Castillo, 2013; Adams et al., 2019).

#### Calculating Sexual Shape Dimorphism (SShD) and Sexual Color Dimorphism (SCoD)

SShD, reflecting the degree of individual development of sexually dimorphic traits at the morphological level, was calculated using shape coordinates aligned through a joint Procrustes fit of female and male facial configurations. Initially, we identified the vector within facial morphospace that links the sex-specific averages. This vector delineates the shape distinctions between males and females. Subsequently, individual SShD scores were determined by projecting each face onto the vector connecting the sex-specific means.

This vector method, i.e., the use of group averages to define an axis of morphological differences between men and women, has been applied in numerous previous studies on human sexual dimorphism (Kleisner et al., 2021; Komori et al., 2011; Mitteroecker et al., 2015; Valenzano et al., 2006).

SCoD was calculated in the same way as SShD, except that instead of using shape coordinates, we used a matrix of skin color measurements, including skin color measured on the cheek and forehead and facial contrast measured around the eye, eyebrow, and lips. First, we defined the values of male and female means and then projected the color measurement of each individual on an axis defined by the sex difference.

The position of an individual’s face (*A*) along an axis connecting male (MM) and female mean (FM) shape/color characteristics can be expressed as a dot product of a vector from the origin to the shape coordinates/color values of *A* and a vector from FM to MM.

#### Ratings of Facial Images

The stimuli were assessed for attractiveness by an unrelated sample of raters, whereby each rater rated a set of portraits of the opposite sex. Participants were recruited mostly via the internet (social media) and then redirected to an online survey platform (Qualtrics.com). Raters viewed each portrait on a computer using a full-screen browser with a survey session. They saw always only one photograph at a time and assessed attractiveness on a 7-point scale (ranging from 1—very unattractive to 7—very attractive). There was no time limit for exposure to each portrait and the order of photographs was randomized for each session.

Facial photographs of Vietnamese men were rated by 124 Vietnamese women (M age = 22.96; SD = 4.26; range = 18–48) and portraits of Vietnamese women were rated by 86 Vietnamese men (M age = 22.2; SD = 3.76; range = 18–47). Each rater evaluated 30 randomly assigned facial portraits out of the dataset. This approach was selected to reduce the usual high attrition of raters due to the long duration of the rating sessions. Cameroonian men were rated by 51 Cameroonian women (M age = 23.37; SD = 4.25; range = 18–45) and Cameroonian women were rated by 49 Cameroonian men (M age = 22.96; SD = 3.23; range = 19–33). Czech men were rated by 80 Czech women (M age = 20.36; SD = 1.70; range = 19–27) and Czech women were rated by 32 Czech men (M age = 21.72; SD = 2.76; range = 19–31). In the Cameroonian and Czech samples, each rater was presented with the full set of photographs (for example: each photograph of a Cameroonian man received 51 ratings).

Excluded from further analysis were the results of raters younger than 18, older than 50, and (self-reported) non-heterosexuals. All participants provided their informed consent by clicking on the “I agree” button to consent with their participation in the study. Interrater agreement using intraclass correlation (ICC, 3k; see Shrout & Fleiss, 1979) was generally high (ICC for all rater datasets > 0.95; for a full overview of raters’ variables see Table S4 in supplementary materials).

### Statistical Analysis

An exploratory analysis of color dimorphism was conducted in SPSS Statistics 20. Color and contrast measurements were compared by a one-way ANOVA. Mean facial values for each color channel (L*a*b*) were tested separately. To account for minor differences in sexual dimorphism, further tests were conducted separately for each measured facial area (forehead, eyebrow area, eye area, cheek, and mouth area). The relationship between perceived attractiveness and components of SCoD was investigated using the Pearson correlation.

We used the *permudist* function from the Morpho package in R (Schlager, 2017) to compare the distances between sex-specific group means in facial shape and color. This was done separately for each of our population samples with a permutation test based on 10,000 replications. The effect size was estimated by Cohen’s *d*.

#### Limitations

A certain limitation of this study is that comparing a total of three distinct populations does not allow for any statistical analysis of the intercultural differences. Our conclusions are therefore based on the observation of the degree and significance of intracultural effects.

Facial skin lightness and coloration are affected by sun exposure. Any sex-related differences in these values, or the lack of these differences, could therefore be the result of behavioral differences between men and women. To evaluate this effect, we measured the base skin color of the Cameroonian and Czech participants with an Ocean Optics Flame-S spectrophotometer. Measurements were taken on the inner side of the arm, where the effect of sun exposure would be minimal, and on the forehead and cheek for reference. The results measured from the inner arm were then compared with the facial measurements taken with the spectrophotometer, as well as measurements from facial photographs (Table S3 in supplementary materials).

Several minor issues arose during data acquisition. They led to slight differences between the samples but the photograph acquisition process in each culture was fully standardized. The levels of sexual dimorphism compared in our study were measured separately for each culture by an intracultural analysis of male and female facial images. It is thus safe to assume that the abovementioned slight differences between the samples had no significant effect on the results.

Some participants had visible scars, shave marks, or lipstick residue, which made it impossible to take certain measurements (e.g., lips region contrast). These individuals were excluded from the relevant parts of the analysis, resulting in varying sample sizes in the correlation table (Table S2 in supplementary materials). Additionally, there is a slight variation in rater counts for the Vietnamese portraits due to the randomization of photos for each rating session coupled with a high rater attrition.

## Results

**Fig. 2:**
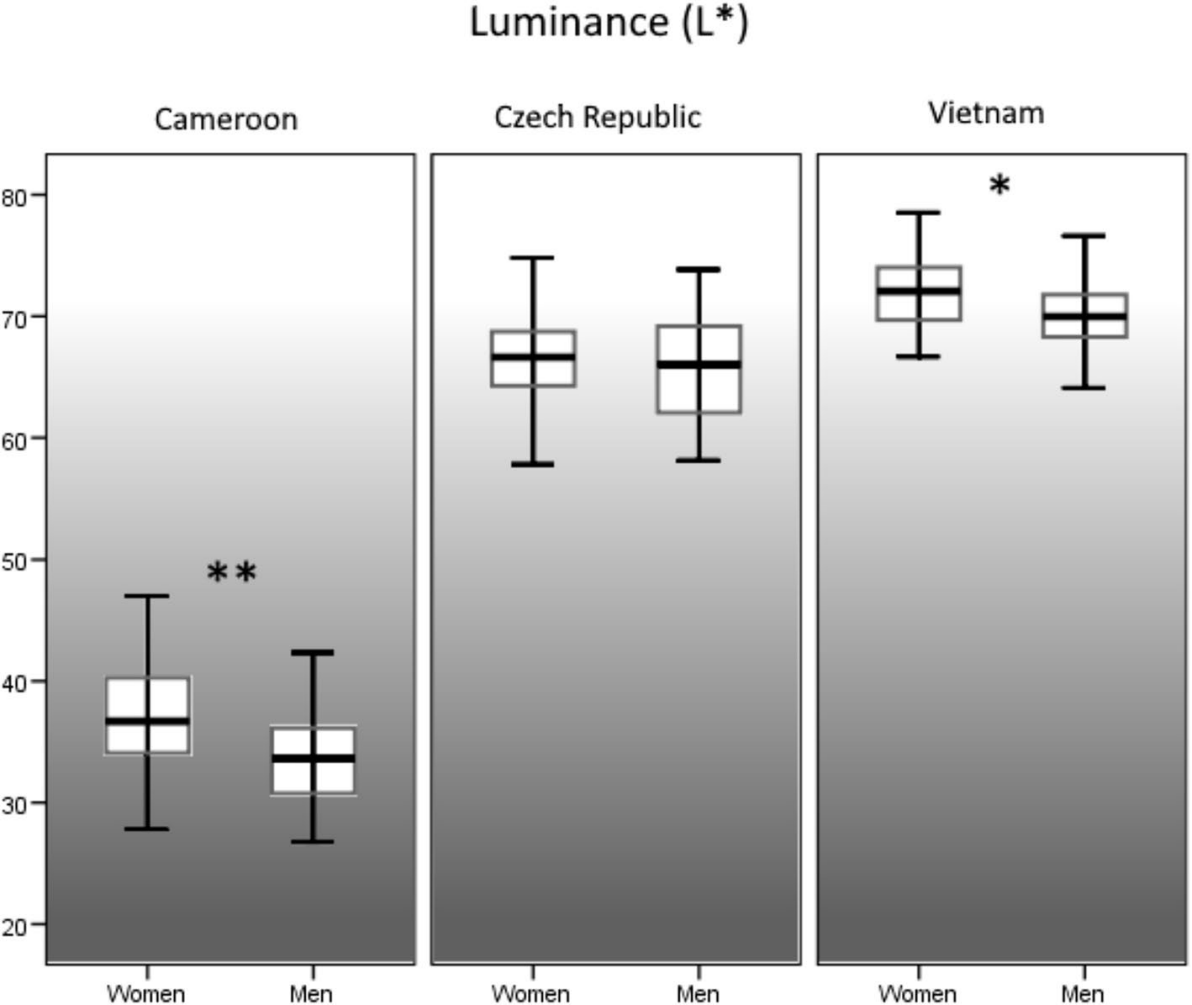
Sexual dimorphism in overall skin luminance (L*). *p < 0.05 **p < 0.01

**Fig. 3:**
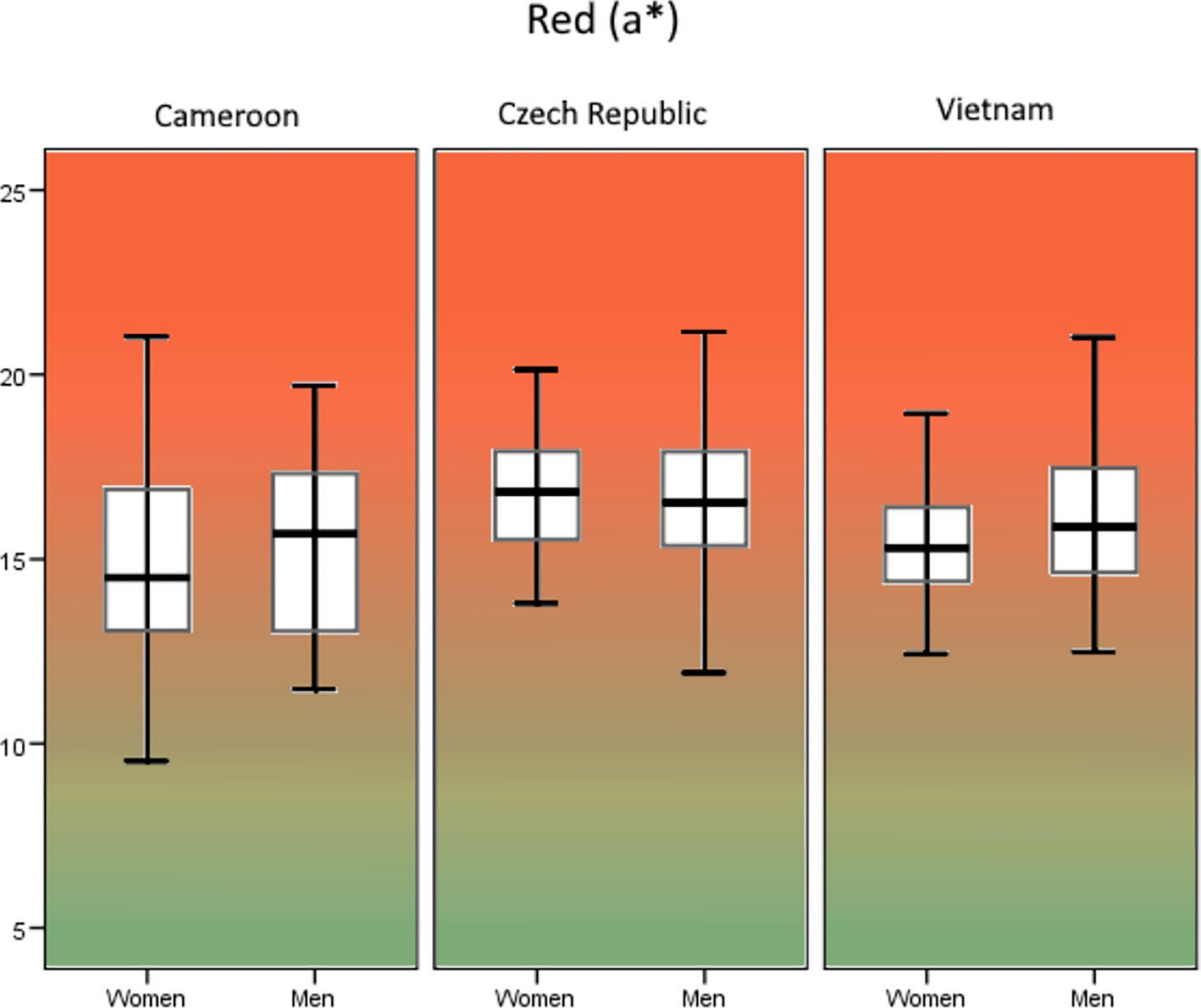
Sexual dimorphism in overall skin redness (a*). *p < 0.05 **p < 0.01 (Color figure online)

**Fig. 4:**
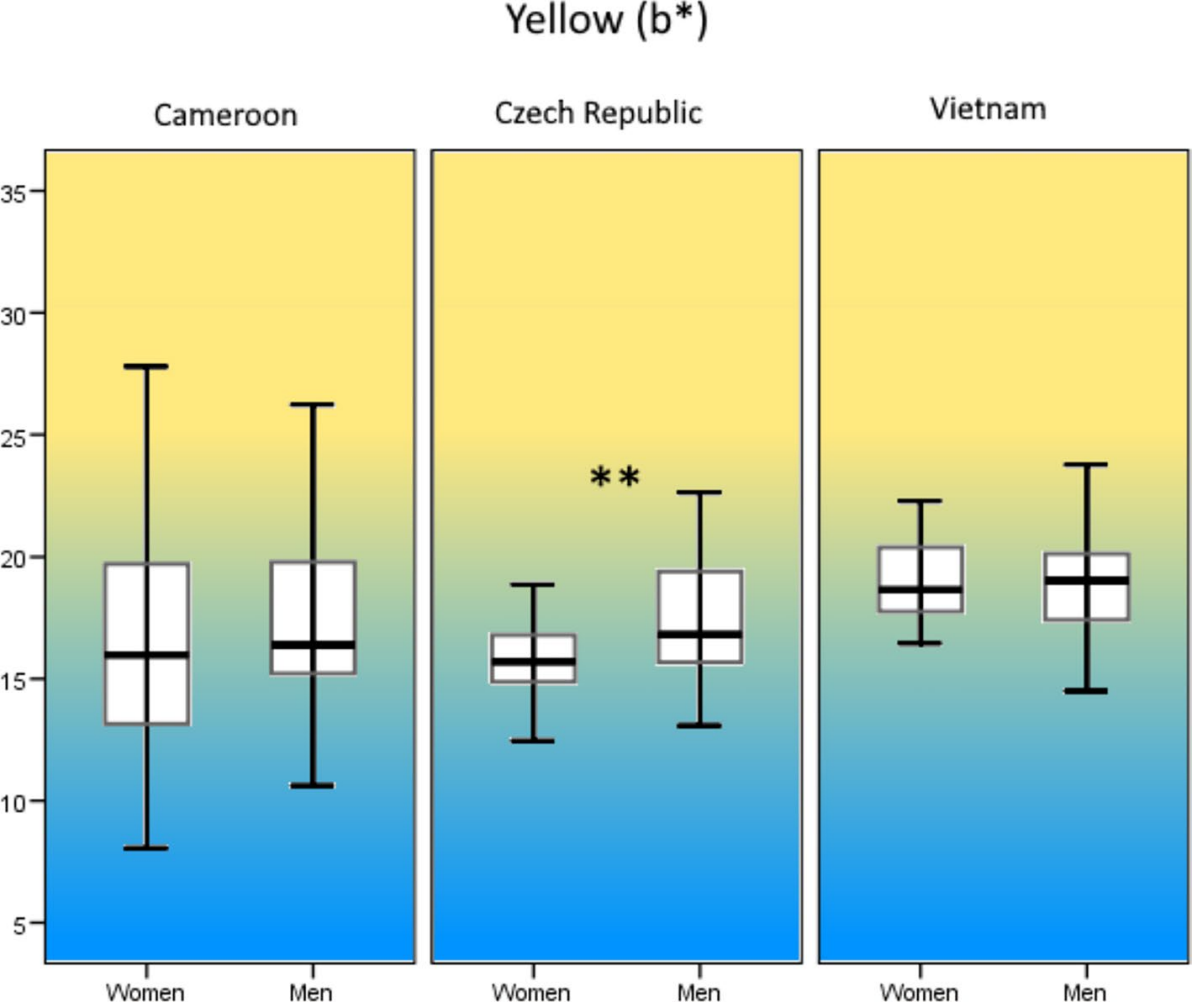
Sexual dimorphism in overall skin yellowness (b*). *p < 0.05 **p < 0.01 (Color figure online)

In the Cameroonian sample, we found a significant dimorphism in skin lightness (*F*[1, 112] = 13.96, *p* < 0.001, *R*^2^ = 0.104, *d* = 0.68, Fig. 2) but no dimorphism in skin redness (*F*[1, 112] = 1.41, *p* = 0.237, *R*^2^ = 0.004, *d* = 0.12, Fig. 3) or skin yellowness (*F*[1, 112] = 1.11, *p* = 0.294, *R*^2^ = 0.001, *d* = 0.06, Fig. 4). In the Czech sample, sexual dimorphism in skin lightness was insignificant (*F*[1, 97] = 0.36, *p* = 0.550, *R*^2^ = 0.007, *d* = 0.16, Fig. 2), we found no sex differences in skin redness (*F*[1, 97] = 1.54, *p* = 0.217, *R*^2^ = 0.006, *d* = 0.15, Fig. 3), but skin yellowness differed significantly between the sexes (*F*[1, 97] = 16.62, *p* < 0.001, *R*^2^ = 0.139, *d* = 0.80, Fig. 4). In the Vietnamese sample, our results show significant sexual dimorphism in skin lightness (*F*[1, 90] = 6.66, *p* = 0. 011, *R*^2^ = 0.059, *d* = 0.50, Fig. 2) but no significant sexual dimorphism in skin redness (*F*[1, 90] = 1.73, *p* = 0.191, *R*^2^ = 0.008, *d* = 0.18, Fig. 3) or skin yellowness (*F*[1, 90] = 0.001, *p* = 0.976, *R*^2^ < 0.001, *d* < 0.0, Fig. 4).

Our findings on facial contrast dimorphism in Cameroonians and Czechs are mostly in line with previous results (Pokorný & Kleisner, 2021), except for the luminance contrast in the eye area, which was in our current Czech sample not statistically significant. The Vietnamese sample showed a pattern similar to the Cameroonian sample, with significant sexual dimorphism in the luminance contrast in the eyebrow (*F*[1, 83] = 24.78, *p* < 0.001, *R*^2^ = 0.223, *d* = 1.07) and eye regions (*F*[1, 88] = 9.20, *p* = 0.003, *R*^2^ = 0.085, *d* = 0.61) but no significant difference in the lips region (*F*[1, 71] = < 1, *p* = 0.924, *R*^2^ < 0.001, *d* = 0.06 see Table S1 in supplementary materials for full results).

In the Cameroonian sample, perceived attractiveness was positively correlated with facial luminance in both men (*r* = 0.28; *p* = 0.043) and women (*r* = 0.34; *p* < 0.01). This effect was not significant in either the Czech (men: *r* = 0.18; *p* = 0.221, women: *r* = − 0.17; *p* = 0.235) or the Vietnamese sample (men: *r* = 0.12; *p* = 0.515, women: *r* = 0.06; *p* = 0.623). For full results on the effect of skin color on perceived attractiveness, see Table S2 in supplementary materials.

**Fig. 5:**
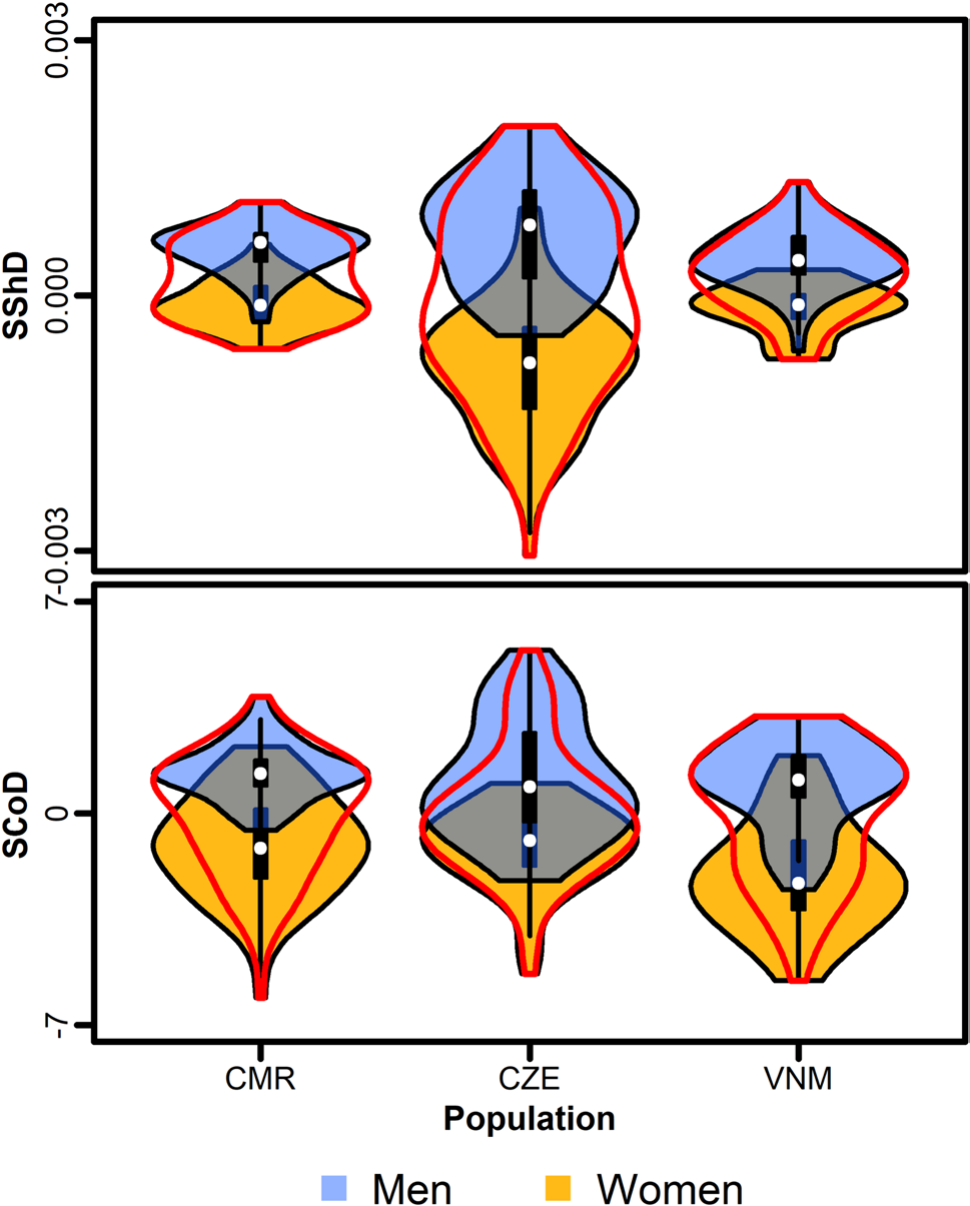
Violin plots showing the levels of sexual dimorphism in shape (SShD) and color (SCoD) in three cultures (CMR—Cameroon, CZE—Czech Republic, VNM—Vietnam). White points indicate medians; black rectangles represent interquartile ranges (Color figure online)

A permutation test based on a random assignment of observations to sex groups showed that sex differences in facial shape expressed by Euclidean distance between male and female means were statistically significant in all three compared populations: Cameroonians (*p* < 0.001, mean distance = 0.02, *d* = 0.50), Czechs (*p* < 0.001, mean distance = 0.04, *d* = 0.73), and Vietnamese (*p* < 0.001, mean distance = 0.02, *d* = 0.57). Levels of SShD varied considerably (Fig. 5): The Czech sample showed a much higher level of SShD than the Cameroonians and Vietnamese samples, which displayed comparable degrees of sex differences in facial morphology.

An analogical comparison of SCoD revealed rather the opposite pattern. The lowest level of SCoD was observed in the Czech sample (*p* < 0.005, mean distance = 1.74, *d* = 0.56), Cameroonians displayed much higher sex differences in color (*p* < 0.001, mean distance = 2.64, *d* = 0.63), and the highest levels of SCoD were found in the Vietnamese sample (*p* < 0.001, mean distance = 3.28, *d* = 0.69); see also Fig. 5. All in all, this suggests a mild pattern of substitution between the levels of SShD and SCoD, where, on average, lower levels of SShD tend to be compensated by higher levels of sex differences in color (SCoD).

## Discussion

The pattern of overall sexual dimorphism, represented by the degree of SShD and various components of SCoD was different in each studied population. The Cameroonian and Vietnamese samples exhibited significant sexual dimorphism in skin luminance: men were on average darker than women, which is assumed to be a universal sexually dimorphic trait (Jablonski & Chaplin, 2000; van den Berghe & Frost, 1986). Conversely, we found in these samples no significant sex differences in skin redness or yellowness. Europeans, represented by a sample of Czechs, did not manifest the expected sex difference in skin luminance. Instead, we observed a significant difference in the yellow-blue channel of the CIELab color space: men were on average yellower than women. Moreover, the level of SShD was in the Czech sample also higher than in the other two studied populations. These aspects of sex-typicality might compensate for the lack of dimorphism in skin luminance and substitute it in the processes of sex recognition and biological quality assessment.

In human populations where the parts of the face which bear sex-specific traits have been subject to some other selective force, sex recognition can be facilitated by other facial regions or types of dimorphism. Furthermore, these secondary sexually dimorphic traits are likely to become exaggerated, because the need for sex recognition creates a constant pressure on maintaining a certain level of sexual dimorphism. Over time, these secondary cues can take on the role of the primary means of sex recognition. In the course of this process, the shape component of sexual dimorphism can expand to compensate for the limited color component—and vice versa. This can lead to different degrees and types of sexual dimorphism among human populations, populations which developed under different selective pressures and constraints.

Such mechanism might explain our recent findings, particularly the pattern of sexual dimorphism in the sample from the Czech Republic: European populations have been under a strong selective pressure to maintain the rate of vitamin D_3_ synthesis in an environment with low UVB radiation (Jablonski & Chaplin, 2010). This led to a decrease of eumelanin levels in the skin and a lighter complexion in modern-day individuals of European descent. The potential for sex differences in skin lightness is thus smaller in populations where eumelanin is maintained at certain levels by the abovementioned constraints. As a result, we would expect to find higher levels of sexual dimorphism in areas unrelated to eumelanin content, for instance in the blue and yellow hues, that is b* in the CIEL*a*b* color space, typically associated with pheomelanin and carotenoids (Ito & Wakamatsu, 2003), or in facial shape (SShD). In the studied European sample (Czech Republic), we have indeed found no sex differences in skin lightness (L*) but observed a significant level of dimorphism in skin yellowness (b*) in favor of males and a higher SShD than in either the African (Cameroon) or the Asian (Vietnam) sample. This interpretation, however, is challenged by our findings from the Vietnamese population, which is also very light-skinned but shows significant skin luminance dimorphism and no dimorphism in the yellow-blue channel. This might be due to cross-populational differences in the pheomelanin content and blood perfusion, but it was not within the scope of this study to compare absolute skin color measurements between the populations.

Similarly, suboptimal ecological conditions may negatively influence male body size subsequently affecting their facial dimorphism (Magid et al., 2018). The harsh tropical environment in Central Africa may have historically hindered the development of masculine facial traits among Cameroonian men. In this context, skin color dimorphism could have served as a less costly means of expressing sex-typical characteristics in the Cameroonian population, as evidenced by the patterns observed in our data. This is further reinforced by a study indicating that the preference for sex-specific morphology is a relatively recent phenomenon, primarily observed in developed, urbanized societies (Scott et al., 2014). If human populations differ in the type and range of sexual dimorphism, as our results suggest, one would expect similar differences in mate choice and attractiveness perception among various populations. Yet although there is a general cross-cultural agreement in attractiveness ratings (Rhodes, 2006; Stephen et al., 2012), certain specific differences seem to support our claim: for instance, Coetzee et al. (2014) have shown that African raters (South Africa) tend to assess female attractiveness based on color cues, while European raters (Scotland) tend to rely more on shape cues. Kleisner et al. (2017) reached a similar result: attractiveness of African women rated by African participants (Cameroon, Namibia) was predicted by color cues (light skin), but when Europeans (Czechs) rated the attractiveness of African women it was not. More recently, Fiala et al. (2022) have shown that color cues affected also perceived sex-typicality in Africans (Cameroon) but not in Europeans (Czech Republic), with the sole exception of skin yellowness: men with yellower skin were perceived as more masculine. Lu et al. (2022) compared various aspects of skin color in Caucasian and Chinese populations and reported a preference for lighter skin among the Chinese but not among Caucasian raters, and a preference for yellower skin in Caucasian but not the Chinese raters. These findings are fully in line with our current observation regarding sexual dimorphism in the shape and color of African and European faces and are further expanded by our results concerning perceived attractiveness. Cameroonians showed a preference for lighter skin tones, while Czechs did not, as would be expected based on the observed SCoD of the respective populations. However, since the preference for lighter skin in the Cameroonian sample was significant for both sexes, this could be as well due to the social connotations of skin color in the population. The introduction of Eurocentric standards of beauty during the colonial period intensified colorism, a discriminatory system based on skin color, in many non-European populations (Hall, 2021; Martinez-Ramirez et al., 2023). The influence of colorism is also evident in Africa, where it was reinforced during the colonial era and has left a lasting impact on society. This has influenced perceptions of social status and local ideals of beauty. Today, the impact of colorism is manifested in the widespread use of skin-bleaching products, driven by the desire to conform to beauty ideals, which poses significant health risks to consumers (Daftary et al., 2023).

Somewhat surprising was the lack of a significant correlation between perceived attractiveness and skin luminance among Vietnamese, as our sample manifested a significant dimorphism in facial luminance. This sexual difference could be, in the case of East-Asians, driven by different needs for vitamin D as proposed by Jablonski and Chaplin (2013), rather than by sexual selection Perhaps other instances of disagreement in attractiveness ratings (e.g., Jones & Hill, 1993; Zebrowitz et al., 2012) could be explained similarly after a multi-level analysis of sexual dimorphism in the studied samples.

Our results contribute to a growing body of evidence which indicates that human sexual dimorphism is a complex and multidimensional phenomenon. The extent and character of sex differences vary widely among human populations and, as our results also indicate, there does not seem to exist any single aspect of sexual dimorphism in human faces that would be universal across all human populations. Nevertheless, a certain level of overall dimorphism is always present and it enables sex recognition. If we were to study sex-typicality based on any single modality, for instance, if we reduced masculinity and femininity to shape measurements alone, we might exclude other, equally important, aspects of sexual differences in another population. Future studies on sexual dimorphism should consider the type and level of dimorphism in both shape (SShD) and color (SCoD), as well as possible natural constraints in the studied populations. Currently, our sample of three populations provides initial insights into this matter but falls short for rigorous statistical analysis of cross-populational differences. We hope consecutive studies exploring a wide variety of distinct populations would offer a means to test our hypothesis.

### Supplementary Information

Below is the link to the electronic supplementary material.

Supplementary file1 (DOCX 55 KB)

## Data Availability

Data are available for public access: https://osf.io/2c6pa/?view_only=67b98517b8cf44bfa993a39e73930adf
