# Supplementary material for: Sexual Dimorphism: The Interrelation of Shape and Color
Source: Arch Sex Behav. 2024 Jun 28;53(8):3255–65. doi: 10.1007/s10508-024-02918-1 (PMC11335828; doi:10.1007/s10508-024-02918-1)
Supplement: Supplementary file 1 — Supplementary file1 (DOCX 55 KB) [file 10508_2024_2918_MOESM1_ESM.docx]

**Supplementary materials**

Table S1: Effect size of sex differences in skin color and facial contrast

|  | overall skin color | | | eyebrow/skin contrast | | | eye/skin contrast | | | lips/skin contrast | | |
| --- | --- | --- | --- | --- | --- | --- | --- | --- | --- | --- | --- | --- |
|  | L* | a* | b* | L* | a* | b* | L* | a* | b* | L* | a* | b* |
| Cameroonians | 13,964** | 1,412 | 1,113 | 41,779** | 5,347* | 0,040 | 12,124** | 8,663** | 44,710** | 0,381 | 7,991** | 3,029 |
| Czechs | 0,360 | 1,543 | 16,627** | 15,095** | 13,969** | 11,703** | 3,438 | 2,494 | 0,980 | 6,494* | 19,859** | 0,590 |
| Vietnamese | 6,667* | 1,736 | 0,001 | 24,782** | 42,852** | 48,999** | 9,201** | 1,263 | 1,368 | 0,009 | 0,032 | 1,530 |

*p<0.05 **p<0.01

Table S2: Correlation coefficients of perceived attractiveness with facial contrast, skin color, and luminance

|  | Cameroon | | Czech Republic | | Vietnam | |
| --- | --- | --- | --- | --- | --- | --- |
|  | Women | Men | Women | Men | Women | Men |
| eyebrow/skin luminance contrast (L*) | -,132 | ,330^*^ | ,081 | ,125 | ,220 | ,168 |
|  | N=63 | N=50 | N=47 | N=50 | N=26 | N=57 |
| eyebrow/skin red-green contrast (a*) | -,273^*^ | ,269 | -,115 | -,001 | -,099 | ,188 |
|  | N=63 | N=50 | N=47 | N=50 | N=26 | N=57 |
| eyebrow/skin yellow-blue contrast (b*) | -,184 | ,234 | -,100 | ,157 | -,125 | ,309^*^ |
|  | N=63 | N=50 | N=47 | N=50 | N=26 | N=57 |
| eye/skin luminance contrast (L*) | ,198 | -,032 | ,241 | ,041 | -,071 | -,021 |
|  | N=63 | N=50 | N=48 | N=50 | N=30 | N=58 |
| eye/skin red-green contrast (a*) | ,077 | ,285^*^ | ,020 | ,155 | ,042 | -,073 |
|  | N=63 | N=50 | N=48 | N=50 | N=30 | N=58 |
| eye/skin yellow-blue contrast (b*) | ,283^*^ | ,270 | ,201 | ,121 | ,299 | ,008 |
|  | N=63 | N=50 | N=48 | N=50 | N=30 | N=58 |
| lips/skin luminance contrast (L*) | ,227 | -,024 | ,117 | -,446^**^ | -,023 | ,200 |
|  | N=63 | N=50 | N=48 | N=48 | N=12 | N=59 |
| lips/skin red-green contrast (a*) | -,077 | -,134 | -,221 | -,183 | -,248 | -,226 |
|  | N=63 | N=50 | N=48 | N=48 | N=12 | N=59 |
| lips/skin yellow-blue contrast (b*) | ,168 | ,030 | ,167 | -,160 | ,201 | ,026 |
|  | N=63 | N=50 | N=48 | N=48 | N=12 | N=59 |
| overall facial luminance (L*) | ,349^**^ | ,287^*^ | ,180 | -,171 | ,122 | ,065 |
|  | N=63 | N=50 | N=48 | N=50 | N=31 | N=59 |
| overall facial redness (a*) | ,176 | ,175 | -,362^*^ | -,208 | -,156 | -,092 |
|  | N=63 | N=50 | N=48 | N=50 | N=31 | N=59 |
| overall facial yellowness (b*) | ,188 | ,205 | -,266 | ,097 | -,097 | ,023 |
|  | N=63 | N=50 | N=48 | N=50 | N=31 | N=59 |

*p<0.05 **p<0.01

Table S3: Correlation coefficients of base skin color measured under the arm (using a spectrophotometer) with facial measurements conducted in vivo (using a spectrophotometer) and from facial photographs (using ImageJ software).

| **L*** |  | Cameroon | Czech Republic |
| --- | --- | --- | --- |
| Spectrophotometer | Forehead luminance (L*) | 0,593** | 0,585** |
|  |  | N=72 | N=98 |
|  | Cheek luminance (L*) | 0,653** | 0,663** |
|  |  | N=72 | N=98 |
| ImageJ | Mean luminance (L*) | 0,634** | 0,229* |
|  |  | N=72 | N=98 |

| **a*** |  | Cameroon | Czech Republic |
| --- | --- | --- | --- |
| Spectrophotometer | Forehead redness (a*) | 0,483** | 0,328** |
|  |  | N=72 | N=98 |
|  | Cheek redness (a*) | 0,634** | 0,361** |
|  |  | N=72 | N=98 |
| ImageJ | Mean redness (a*) | 0,523** | 0,217* |
|  |  | N=72 | N=98 |

| **b*** |  | Cameroon | Czech Republic |
| --- | --- | --- | --- |
| Spectrophotometer | Forehead yellowness (b*) | 0,638** | 0,588** |
|  |  | N=72 | N=98 |
|  | Cheek yellowness (b*) | 0,719** | 0,569** |
|  |  | N=72 | N=98 |
| ImageJ | Mean yellowness (b*) | 0,752** | 0,444** |
|  |  | N=72 | N=98 |

*p<0.05 **p<0.01

Table S4: Overview of rater variables

| Stimuli |  | Number of Raters per Photograph | Mean Age | SD Age | Age Range | Sex of Raters | Intraclass Correlations Coefficient  (3,k)* |
| --- | --- | --- | --- | --- | --- | --- | --- |
| Cameroon | Women | 49 | 22.96 | 3.23 | 19-33 | Male | 0.96 |
|  | Men | 51 | 23.37 | 4.25 | 18-45 | Female | 0.96 |
| Czech Republic | Women | 32 | 21.72 | 2.76 | 19-31 | Male | 0.97 |
|  | Men | 80 | 20.36 | 1.70 | 19-27 | Female | 0.99 |
| Vietnam | Women | 83 | 22.20 | 3.76 | 18-47 | Male | 0.96 |
|  | Men | 62 | 22.96 | 4.26 | 18-48 | Female | 0.98 |

*Average, fixed raters ICC
